# Depression and Antidepressants During Pregnancy: Craniofacial Defects Due to Stem/Progenitor Cell Deregulation Mediated by Serotonin

**DOI:** 10.3389/fcell.2021.632766

**Published:** 2021-08-12

**Authors:** Natalia Sánchez, Jesús Juárez-Balarezo, Marcia Olhaberry, Humberto González-Oneto, Antonia Muzard, María Jesús Mardonez, Pamela Franco, Felipe Barrera, Marcia Gaete

**Affiliations:** ^1^Department of Anatomy, Faculty of Medicine, Pontificia Universidad Católica de Chile, Santiago, Chile; ^2^Department of Psychology, Pontificia Universidad Católica de Chile, Santiago, Chile; ^3^Millennium Institute for Research in Depression and Personality (MIDAP), Santiago, Chile; ^4^School of Dentistry, Faculty of Medicine, Pontificia Universidad Católica de Chile, Santiago, Chile

**Keywords:** craniofacial defects, antidepressant, depression, pregnancy, stem cells

## Abstract

Depression is a common and debilitating mood disorder that increases in prevalence during pregnancy. Worldwide, 7 to 12% of pregnant women experience depression, in which the associated risk factors include socio-demographic, psychological, and socioeconomic variables. Maternal depression could have psychological, anatomical, and physiological consequences in the newborn. Depression has been related to a downregulation in serotonin levels in the brain. Accordingly, the most commonly prescribed pharmacotherapy is based on selective serotonin reuptake inhibitors (SSRIs), which increase local serotonin concentration. Even though the use of SSRIs has few adverse effects compared with other antidepressants, altering serotonin levels has been associated with the advent of anatomical and physiological changes *in utero*, leading to defects in craniofacial development, including craniosynostosis, cleft palate, and dental defects. Migration and proliferation of neural crest cells, which contribute to the formation of bone, cartilage, palate, teeth, and salivary glands in the craniofacial region, are regulated by serotonin. Specifically, craniofacial progenitor cells are affected by serotonin levels, producing a misbalance between their proliferation and differentiation. Thus, it is possible to hypothesize that craniofacial development will be affected by the changes in serotonin levels, happening during maternal depression or after the use of SSRIs, which cross the placental barrier, increasing the risk of craniofacial defects. In this review, we provide a synthesis of the current research on depression and the use of SSRI during pregnancy, and how this could be related to craniofacial defects using an interdisciplinary perspective integrating psychological, clinical, and developmental biology perspectives. We discuss the mechanisms by which serotonin could influence craniofacial development and stem/progenitor cells, proposing some transcription factors as mediators of serotonin signaling, and craniofacial stem/progenitor cell biology. We finally highlight the importance of non-pharmacological therapies for depression on fertile and pregnant women, and provide an individual analysis of the risk–benefit balance for the use of antidepressants during pregnancy

## Introduction

Maternal depression is one of the most frequent mood disorders occurring during and after pregnancy, affecting 7–12% of women in developed countries (Charlton et al., 2015; Huybrechts et al., 2015; Fairbrother et al., 2017; Field, 2017a; McAndrew, 2019). The depressive symptomatology during pregnancy has been identified as a predictor for postnatal depression (Field, 2011; Koutra et al., 2014; Raskin et al., 2016). On the other hand, the development of the fetus is affected by maternal depression, being correlated with fetus low heart rate baseline, premature births, protracted descent (Emory and Dieter, 2006), and low size and weight of the newborns (Field, 2011; Dadi et al., 2020; Hompoth et al., 2020). Also, the offspring of depressed mothers has a high risk of depression (Pawlby et al., 2009) and negative consequences in affective, cognitive, and behavioral development (Grace et al., 2003; Milgrom et al., 2008; Pearson et al., 2012).

The identification of risk factors associated with maternal depression can contribute to their prevention. The risk factors can be classified as prenatal factors, factors related to pregnancy, and factors related to the mother herself. Overall, the lack of a partner, absence of socio-familiar support network, low income, insecurity attachments, history of depression, and extreme ages (teenagers or>40 years), are prenatal risk factors for maternal depression (Faisal-Cury and Rossi Menezes, 2007; Olhaberry et al., 2014; Field, 2017b). Pregnancy-related risk factors are the lack of pregnancy planning, undesired pregnancy, and the ambivalence about maternity (Bowen and Muhajarine, 2006). Maternal-related risk factors include stress, drug consumption, violence, conflicts with partners, low educational level (Escribe-Aguir et al., 2008; Field, 2017b), insecure attachment to their own mother (Murray et al., 1996; Bifulco et al., 2006), and adverse or traumatic experiences during childhood and adolescence (Buist and Janson, 2001; Nelson et al., 2002).

The treatment for depression usually includes psychotherapy, pharmacotherapy, or a combination of both. Regarding psychotherapy, cognitive–behavioral therapy has been demonstrated to be effective in the decrease of symptoms and remission of depression during pregnancy (O’Connor et al., 2016). Nevertheless, the adherence to psychotherapy is difficult (Rojas et al., 2015), and the outcome depends on the individual traits of the mothers (Miranda et al., 2017). In the recent years, new behavioral interventions have emerged as an alternative treatment for maternal depression, such as interpersonal psychotherapy, mindfulness, peer support groups, massage, tai chi, yoga, aerobic exercise, and sleep interventions (Field, 2017b; Ladyman et al., 2020; Lucena et al., 2020).

Pharmacotherapy is frequently used to treat depression: approximately one-third of pregnant depressive women use antidepressants (Ramos et al., 2007; Goodman and Tully, 2009; Jimenez-Solem et al., 2013; Molenaar et al., 2020). In line with the serotonergic theory of depression, which proposes that diminished activity of serotonin pathways plays a causal role in the pathophysiology of depression (Kerr, 1994), the most commonly prescribed antidepressants belong to the family of the selective serotonin reuptake inhibitors (SSRIs). SSRIs act by blocking the serotonin transporter (SERT), preventing serotonin recapture, which increases the extracellular concentration of physiologically released serotonin (Stahl, 1998; Gershon and Tack, 2007). Fluoxetine, sertraline, and citalopram are the most prescribed SSRIs (Kern et al., 2020; Molenaar et al., 2020).

Apart from its role as a neurotransmitter related to mood, serotonin appears to have a relevant role during development (Shuey et al., 1993; Buznikov et al., 2001; Kaihola et al., 2016). From this, the question about whether depression or antidepressants interfere with developmental process during pregnancy emerges. As the abovementioned effects of maternal depression at birth, negative effects of the use of antidepressants during pregnancy have been reported, including non-optimal birth outcomes (i.e., preterm delivery and lower Apgar scores), persistent pulmonary hypertension of the newborn, neonatal withdrawal/toxicity syndrome, greater internalizing behaviors at toddler age, and greater risk for autism spectrum disorder (Meltzer-Brody et al., 2011; Huybrechts et al., 2015; Field, 2017b). Regarding the craniofacial region, the use of SSRI has also been associated with bone defects like craniosynostosis and dental malformations, affecting mainly the proliferation and differentiation equilibrium in progenitor cells, as described in different experimental models (Shuey et al., 1992; Moiseiwitsch et al., 1998; Cray et al., 2014; Calibuso-Salazar and Ten Eyck, 2015; Durham et al., 2019), and associated with an increased risk of craniofacial malformations in humans (Alwan et al., 2007; Berard et al., 2015, 2017; Reefhuis et al., 2015; Gao et al., 2018).

Depression and the use of SSRIs have increased over the last few years (Global Burden of Disease Study, 2017). Therefore, it is necessary to build a systematic model to allocate the current knowledge that links depression, SSRI treatment, and craniofacial development. In this review, we performed a bibliographic search using search engines such as PubMed and Google Scholar, looking for cellular, animal, and human research that associates the role of serotonin during craniofacial development with maternal depression or the use of antidepressants. To provide a background to understand this topic, we primarily will describe craniofacial development and the general origin of craniofacial defects, to introduce then the role of serotonin in the craniofacial region development, describing the craniofacial defects related to the use of SSRI. We finally propose a model to explain how depression or antidepressants, as environmental factors, could generate craniofacial developmental defects in the offspring, by altering the stem/progenitor cell biology.

## Craniofacial Development and the Origin of Craniofacial Defects

Human craniofacial congenital defects vary between 1 and 4% in different countries (Dolk, 2005; Canfield et al., 2006) having serious functional, aesthetic, and social consequences. This makes it relevant to identify the developmental processes involved in craniofacial congenital defects, and how genetic and environmental factors can alter them.

**FIGURE 1:**
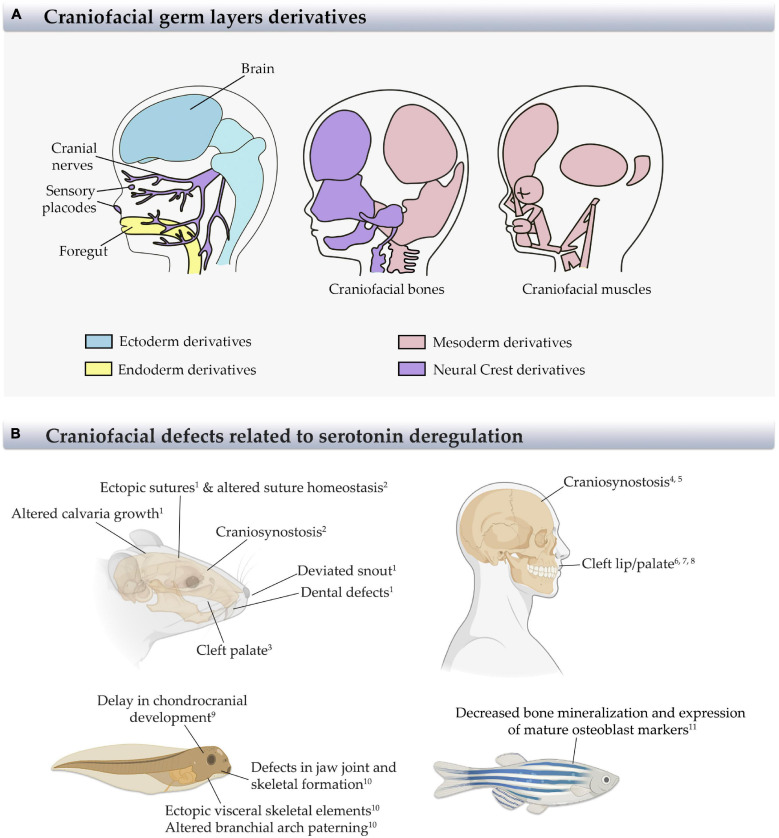
Derivatives structures from germ layers and craniofacial defects in different animal models and humans. **(A)** Scheme of the craniofacial derivatives from ectoderm, mesoderm, endoderm, and neural crest (NCs) showing the main craniofacial structures and the germ layer from which they came. Modified from Carlson (2019). **(B)** Scheme of animal models and humans indicating the most relevant craniofacial defects generated by serotonin deregulation, as reported. References: ^1^Cray et al., 2014; ^2^Durham et al., 2019; ^3^Cabrera et al., 2020; ^4^ Berard et al., 2017; ^5^Alwan et al., 2007; ^6^Colvin et al., 2011; ^7^Louik et al., 2007; ^8^Malm et al., 2011; ^9^Calibuso-Salazar and Ten Eyck, 2015; ^10^Reisoli et al., 2010; ^11^Fraher et al., 2016.

Vertebrate craniofacial development is characterized by a rich crosstalk between the three germ layers and neural crest-derived cells (NCCs) (Couly et al., 2002; Rinon et al., 2007; Grenier et al., 2009; Marcucio et al., 2011; Figure 1A). During development, NCCs show multipotency (stemness) and migratory capabilities (Abzhanov et al., 2003; Adameyko and Fried, 2016). They delaminate alongside the edge of the neural plate and populate the craniofacial region, forming the progenitors for most facial bones, cartilages, salivary glands, and dental mesenchyme at the craniofacial region (Le Douarin et al., 2004). The defects in generation, migration, and differentiation of NCCs could generate a variety of apparently non-related diseases named neurocristopathies (Bolande, 1974). The advance of research and general understanding of NCC development in recent years has led to an increase in the number of reported neurocristopathies (Bolande, 1997; Sato et al., 2019).

Regarding craniofacial tissues, among the malformations typically manifested at birth are maxillary, zygomatic, and mandibular hypoplasia, cleft palate, and auricular defects. The etiology of neurocristopathies includes genetic mutations in the genes *Tcof1* and *Polr1* in Treacher–Collins syndrome, *Sox9* in Pierre Robin sequence, *Sox10* in Waardenburg syndrome, and a region on chromosome 14q32 in Goldenhar syndrome. Additionally, environmental factors such as alcohol, folic acid deficiency, maternal diabetes, infection, and pharmaceutical agents, and their interaction with genetic mutations, have also been related to the development of neurocristopathies (reviewed in Sato et al., 2019; Figure 1B).

The growth of the skull is also an important process during craniofacial development. The skull sutures, zones in which flat bones contact, ossify during the first two postnatal decades, allowing the growth and expansion of the brain. When an imbalance between proliferation and differentiation in the suture cells and adjacent bones occurs, premature bone differentiation leads to premature closure of sutures and produces craniosynostosis (Twigg and Wilkie, 2015). Most of the craniosynostosis alter the shape of the skull, generating a secondary effect such as altered intracranial pressure, blindness, cognitive disabilities, and mental retardation; therefore, surgery is required as treatment (Durham et al., 2019). Craniosynostosis has a prevalence of 1:1,800–2,500 births, being associated to some genetic mutations (*Cdc45*, *Twist*, *Fgfr*, and *Tcf12*) and/or environmental factors including nicotine, hyperthyroidism in pregnant women, and importantly, the use of antidepressants (Twigg and Wilkie, 2015; Durham et al., 2017). The relationship between these environmental factors and craniosynostosis has been described in animal models, which show an altered proliferation and differentiation of stem/progenitor cells, and in humans, in which the newborns from mothers exposed to these disturbances have an increase in craniosynostosis prevalence (Shuey et al., 1992; Carmichael et al., 2008; Grewal et al., 2008; Browne et al., 2011; Berard et al., 2015, 2017; Durham et al., 2017, 2019; Figure 1B).

As the overall growth of the skull is important during craniofacial development, two characteristic craniofacial organs that can be affected during development and are extensively studied are teeth and salivary glands. Teeth and salivary gland development require a tight communication between the oral epithelium and the surrounding NCC-derived mesenchyme. Teeth develop through different stages including the initiation stage, bud stage, cup stage, bell stage, and posterior root formation (Ruch et al., 1995; Thesleff and Sharpe, 1997; Thesleff, 2003). During each stage, defects in the correct sequence of events that will form the teeth produce malformations such as agenesia, hypodontia, or tooth shape abnormalities, which can be presented alone or as part of a major syndrome. Similar to other craniofacial defects, teeth defects are related to well-characterized genetic mutations (*Msx1*, *Pax9*, *Axin2*, *Eda*, W*nt10A*, *Foxc1*, and *Pitx2*, among others) and/or environmental factors like ingestion of chemical substances (fluorides, tetracyclines, dioxins, and thalidomide), malnutrition, vitamin D deficiency, bilirubinemia, thyroid, and parathyroid disturbances, maternal diabetes, severe infections, and metabolic disorders (Brook, 2009; Klein et al., 2013; Figure 1B). As is observed for teeth, salivary gland development also proceeds through different stages: placode, bud, pseudoglandular, canalicular, and cytodifferentiation (Affolter et al., 2003; Patel et al., 2006; Knosp et al., 2012; Hauser and Hoffman, 2015; Chatzeli et al., 2017; Emmerson et al., 2017). Congenital genetic and/or environmental-caused defects during salivary gland development generate aplasic or ectopic glands, mainly associated to syndromes as Levy–Hollister syndrome, oculo–auriculo–vertebral spectrum (OAVS), Treacher–Collins syndrome, and Down syndrome (Togni et al., 2019).

In summary, craniofacial development is a highly sensitive process that occurs early during gestation. Congenital craniofacial defects are multifactorial and are associated with diverse genetic, environmental factors, and the interaction of both (Murray, 2002; Murray and Marazita, 2013; Nagy and Demke, 2014; Durham et al., 2017). Though most of the current research have focused on genetic factors, environmental factors need to be studied as well.

## Serotonin Signaling Components are Present in Craniofacial Tissues

Serotonin is a monoamine synthesized intracellularly from L-tryptophan, released, and later degraded via monoamine oxidase action (Kirk et al., 1997; Sahu et al., 2018). The serotonin signaling is transduced to subcellular events by specific membrane receptors of different classes. Most of the serotonin receptors belong to the superfamily of G-protein-coupled receptors containing a predicted seven-transmembrane domain structure, coupled with Gαi, Gαq/11, or Gαs, given a plethora of biochemical pathways that could be influenced by serotonin receptor activation (Peroutka, 1994; Sahu et al., 2018). Conversely, the serotonin-3 receptor is a ligand-gated ion channel (Hoyer et al., 2002; Millan et al., 2008; Ori et al., 2013). Furthermore, serotonin can act intracellularly after being internalized by SERT or transported through the gap junction between neighboring cells. Then, it can act in two ways: binding to proteins such as Mad3 (protein related to checkpoint in cell division) and serotonin-2 receptor, or by serotonylation of several molecules (covalent addition of serotonin to glutamine residues) [reviewed in Berard et al. (2019)].

Serotonin controls a broad spectrum of biological process, including gastrointestinal motility and secretion, cardiovascular regulation, hemostatic processes, circadian rhythms, sleep–wake cycle, memory, and learning, perception of pain, and appetite and sexual behavior [reviewed in Berger et al. (2009)]. In the nervous system, serotonin has a well-known role as a neurotransmitter, whose imbalance is associated with human psychiatric disorders like depression, anxiety, obsessive–compulsive disorders, autism, and schizophrenia. The brain serotonin is mainly produced by neurons of the raphe nuclei and the pineal gland, in the latter, as a precursor of melatonin. Besides the brain, serotonin is produced by almost all cells, being enriched in the enterochromaffin and myenteric cells of the gut, representing about 95% of the total serotonin secretion (Tsapakis et al., 2012; Sahu et al., 2018).

**FIGURE 2:**
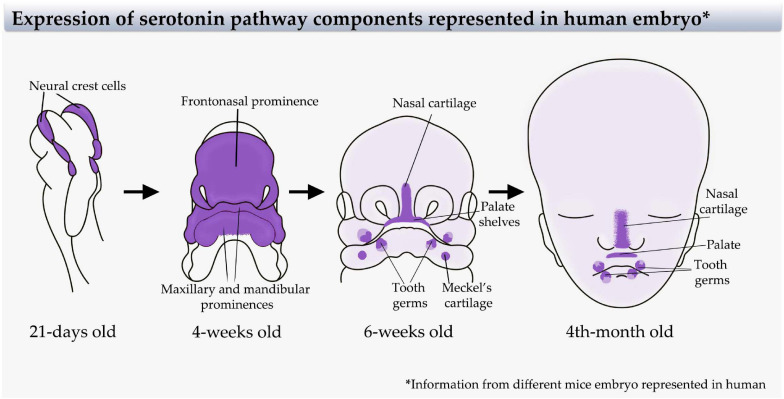
Serotonin pathway component expression in the craniofacial region represented in the human embryo. Sites of serotonin signaling components expression [receptors and serotonin transporter (SERT)] extrapolated from mice embryo animal model (Moiseiwitsch and Lauder, 1995, 1996; Moiseiwitsch et al., 1998; Lauder et al., 2000) to a human embryo. *Left-to-right:* A 21-day-old human embryo (representing the information from E9.5 mice embryo) showing expression in neural crest cells. A 4-week-old human embryo (representing the information from E11.5 mice embryo) showing expression in the first pharyngeal arch and frontonasal prominence. A 6-week-old human embryo (representing the information from E13.5 mice embryo) showing specific expression in the tooth germ, palate, first arch cartilage, and nasal cartilage, and a wide light color representing the expression in the developing craniofacial skeleton. A 4-month-old human embryo (representing the information from E16.5 mice embryo) showing expression in tooth germ, palate, nasal cartilage, and wide light expression in the craniofacial skeleton. Modified from Adameyko and Fried (2016).

In parallel to these roles in metabolism, serotonin has been implicated in several early developmental processes before the onset of neurogenesis, acting as a morphogen that regulates cell proliferation, migration, and differentiation. Some of the processes regulated by serotonin include left–right asymmetry (Levin et al., 2006), neural crest cell formation and migration (Moiseiwitsch and Lauder, 1995; Vichier-Guerre et al., 2017), and heart, bone, and craniofacial development (Shuey et al., 1992, 1993; Yavarone et al., 1993; Moiseiwitsch and Lauder, 1996). In mammals, serotonin required for early development is produced by the embryo, as early as the two-cell stage (Amireault and Dube, 2005; Dube and Amireault, 2007; Kaihola et al., 2016), and from a source supplied by the maternal blood (Cote et al., 2007) and trophoblast placental cells (Bonnin and Levitt, 2011; Kaihola et al., 2015). Uptake of serotonin has been observed in cranial mesenchyme, heart, liver tissues, and, importantly, in migrating neural crest cells (Lauder and Zimmerman, 1988; Narboux-Neme et al., 2008; Vichier-Guerre et al., 2017; Figure 2).

**FIGURE 3:**
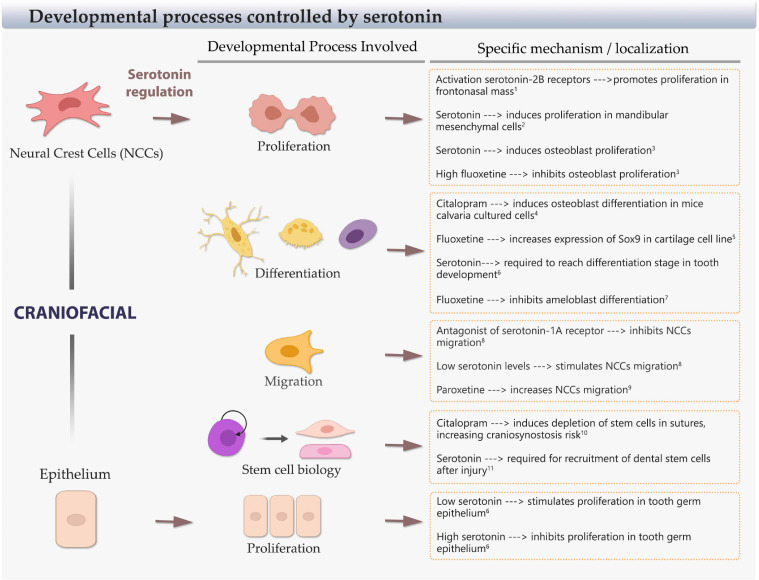
Cellular developmental processes regulated by serotonin. Scheme of craniofacial neural crest cells (NCCs) and epithelial cells, and the main cellular processes controlled by serotonin: proliferation, differentiation, migration, and stem cell balance. The specific regulation, effect, and craniofacial territory/type of cell involved are included. References: ^1^Bhasin et al., 2004; ^2^Buznikov et al., 2001; ^3^Gustafsson et al., 2006; ^4^Cray et al., 2014; ^5^Miyamoto et al., 2017;^6^ Moiseiwitsch and Lauder, 1996; ^7^Riksen et al., 2010; ^8^Moiseiwitsch and Lauder, 1995; ^9^Vichier-Guerre et al., 2017; ^10^Durham et al., 2019; ^11^Baudry et al., 2015.

Interestingly, in the craniofacial region, serotonin receptors are expressed at early stages, and their activation or inactivation are related with several developmental processes. In NCC explants and mouse embryos, the addition of an antagonist of the serotonin-1A receptor inhibited the migration of cranial NCCs (Moiseiwitsch and Lauder, 1995). In whole mouse embryo cultures, blocking the serotonin-2 receptor generates malformed embryos (Choi et al., 1997; Lauder et al., 2000; Bhasin et al., 2004), and in *Xenopus laevis*, it perturbs the development of the heart, face, and eyes (Reisoli et al., 2010). In embryonic mouse mandibular mesenchyme and explant cultures, antagonists for serotonin-2 and -3 receptors block the effects of serotonin on the expression of mandibular proteins (Moiseiwitsch and Lauder, 1997; Figure 3). Concomitantly, SERT is expressed in different regions of the mouse and rat craniofacial mesenchyme and cartilage from E14 to at least E18 (Moiseiwitsch and Lauder, 1997; Moiseiwitsch et al., 1998; Hansson et al., 1999; Lauder et al., 2000; Narboux-Neme et al., 2008; Figure 2). Similarly, sites of serotonin uptake and degradation are identified in mouse tooth germ (Lauder and Zimmerman, 1988; Shuey et al., 1992), and serotonin receptors are expressed in the epithelium of the tooth germ from the bud stage (Moiseiwitsch et al., 1998; Lauder et al., 2000). Serotonin synthesis and uptake have also been detected in palate shelves during palate formation (Wee et al., 1981; Zimmerman et al., 1981; Hirata et al., 2018; Figure 2).

The early expression of the serotonin pathway components and the developmental defects that produce their downregulation or upregulation, including the use of SSRIs, strongly indicate a role of serotonin in the development of craniofacial structures. This makes relevant to understand which cells are affected and what the underlying mechanism implied is.

## Serotonin Has a Role Over Stem/Progenitor Cells that Influences Craniofacial Development

Different studies have shown that serotonin works as a dose-dependent growth regulatory signal for craniofacial progenitor cells. In neural crest mice explants (E9) and dissociated mandibular cells (E12), low levels of serotonin stimulate the migration of NCCs, mediated by serotonin-1A receptor. On the contrary, at high doses, serotonin inhibits the migration of less motile mandibular mesenchymal stem cells (MSCs) (Moiseiwitsch and Lauder, 1995). Similarly, the treatment with paroxetine (SSRI) in NCCs differentiated from human embryonic stem cells triggers an increased proliferation, migration, and AP2-α expression, an important gene involved in the bone plate fusion process in the skull. On the contrary, sertraline decreases the NCC proliferation and increases the expression of AP2-α, demonstrating that SSRIs alter the normal behavior of NCCs (Vichier-Guerre et al., 2017; Figure 3 and Table 1).

**TABLE 1:** Summary of main findings of research relating serotonin signaling pathway disruption and craniofacial development related effects.

Tissue Affected	Type of serotonin signaling disruption	Organ/tissue/cell effect	Involved/affected factors	Model of study	Main methods	References
Bone (skull) and tooth	SSRI exposure	Smaller skull, shorter and narrower snout, skull ectopic suture, fusion of maxillary incisor and absence of root	FGFs	Mouse new-born	*In utero* exposition (E13 to E20) to SSRI. Analysis at P15. Citalopram dosage: 500 μg/day in drinking water.	Cray et al., 2014
Bone (skull)	SSRI exposure	Increased risk of craniosynostosis by depleting calvaria Gli1^+^ stem cells	Gli1^+^ calvaria stem cells	Mouse new-born	*In utero* exposition (E13 to E20) to SSRI. Analysis at P15. Citalopram dosage: 500 μg/day in drinking water.	Durham et al., 2019
Bone (Operculum)	SSRI exposure	Decreased bone mineralization and osteoblast-specific markers expression during embryogenesis. Reduced expression of osteoblast activity markers in cell culture	Runx2	Zebrafish embryo/hMSCs	SSRI exposition at 36 hpf - 130 hpf/hMSC 7d culture. Dosage: Citalopram 15 μM; Sertraline 30 μM.	Fraher et al., 2016
Bone (cell line)	SSRI exposure/Serotonin addition	Serotonin and low dose of SSRI promotes osteoblast proliferation. At high doses of SSRI proliferation is inhibited	Serotonin-2 receptor	MC3T3-E1 (murine pre-osteoblasts)	Fluoxetine dosage: 1 nM, 10 nM, 100 nM, 1 μM, 10 μM Serotonin addition: 1 nM, 10 nM, 100 nM, 1 μM, 10 μM, 50 μM	Gustafsson et al., 2006
-	SSRI exposure	Changes in migration and expression of bone plate fusion-associated factors	AP2-α	ESCs-derived NCCs	Paroxetine and sertraline dosage: 30 nM, 300 nM, 3 μM	Vichier-Guerre et al., 2017
Cartilage (Cell line and knees)	SSRI exposure	Increased Sox9 and decreased Axin2 and Mmp13 expression. Pro-chondrogenic effect on osteoarthritic (OA) model	Sox9	ATDC5/Osteoarthritic-induced rats	OA phenotype induced through surgical meniscus destabilization. OA model fluoxetine dosage: 50 μM, 100 μM, 200 μM injections Cell culture fluoxetine dosage:1 μM, 5 μM, 10 μM	Miyamoto et al., 2017
Hippocampus	SSRI exposure	Increased proliferation in hippocampal progenitors with reduced self-renewal and division	Sox2	Btg1 KO mouse (adult)	Fluoxetine (10 μM) injected intra peritoneal administered for 21 days, since 56 days or 15 months of age.	Micheli et al., 2018
-	Serotonin-2B receptor gain and loss of function	Loss of function leads to a loss of the jaw joint and altered first brachial arch. Gain of function produces abnormal craniofacial development	Serotonin-2B receptor	*Xenopus laevis* embryo	Loss of function viaserotonin-2B receptor morpholino. Gain of function using *in vitro* synthesized serotonin-2B receptor mRNA. Both microinjected at 2-4 cell stage embryos	Reisoli et al., 2010; Ori et al., 2013
Mandibular epithelium	SSRI exposure	Craniofacial defects: maxilla deficiency, absence of lens invagination and open cranial neural folds	-	Mouse embryo	E9 embryos were culture for 48h in presence of SSRI. Dosage: Fluoxetine 1 μM, 10 μM; Sertraline 5 μM, 10 μM, 20 μM.	Shuey et al., 1992
Tooth germ	SSRI exposure/Serotonin addition	Serotonin addition promoted transition between developmental stages.	-	Mouse embryo	E13 embryos mandible explants were culture up to 2 or 8 days. Fluoxetine dosage: 10 μM Serotonin addition: 10 nM, 1 μM, 100 μM	Moiseiwitsch and Lauder, 1996
Craniofacial mesenchyme and epithelia	Serotonin-2B receptor antagonists	Craniofacial malformations as hypoplastic forebrain/frontonasal process, hypoplastic maxilla/mandible, lack of lens invagination and neural tube defects	Serotonin-2B receptor	Mouse embryo	E9 embryos were culture for 48 h in presence of antagonists. Receptor Antagonists: Mianserin (1 μM, 10 μM), Ritanserin (0.1 μM, 1 μM), Ketanserin (1 μM, 10 μM)	Lauder et al., 2000
Craniofacial bone and cartilage	SSRI exposure	Delayed development of frontoparietal bones, mandible, nasal cartilage, and squamosal bones.	-	*Eleutherodactylus coqui* embryo	Embryos were culture from TS1 to TS15 (Townsend and Stewart staging). Fluoxetine dosage: 100 μM, 250 μM, 500 μM, 1 mM	Calibuso-Salazar and Ten Eyck, 2015
Mandible	Serotonin-1A/2A-2C/3 receptors antagonists and serotonin addition	Serotonin addition stimulated tooth germ development at bud and bell stages. The same effect was inhibited by serotonin-1A receptor antagonist and reversed by the serotonin-3 receptor antagonist.	Serotonin-1A/2A-2C/3 receptors	Mouse embryo	E13 embryos mandible explants were culture up to 8 days. Serotonin addition: 10 nM, 1 μM, 100 μM. Receptor Antagonists (10 μM): NAN-190, Mianserin, Zofran	Moiseiwitsch et al., 1998
Dental pulp	Serotonin-2B/7 receptors antagonists	Altered tooth reparative process upon disrupted injury signals	Serotonin-2B/7receptors	Rat dental pulp injury	Gelatine hydrogel microspheres loaded with antagonists were implanted within the pulp just after lesion and followed up to 30 days. Receptor Antagonists (100nM): RS127445 (serotonin-2B receptor), SB269970 (serotonin-7 receptor)	Baudry et al., 2015
Ameloblast-like cell line	SSRI exposure/serotonin addition	Serotonin and SSRI treatment downregulated amelogenin, enamelin and MMP20 expression, as well as VEGF, MCP-1, and IP-10. Also, both treatments enhanced alkaline phosphatase activity	-	LS8	Cell cultures were analyzed at 1, 3 and/or 7 days. Fluoxetine dosage: 0.1 μM, 1 μM, 10 μM Serotonin addition: 0.1 μM, 1 μM, 10 μM	Riksen et al., 2010
Cranial neural crest and mandibular mesenchyme	Serotonin and serotonin-1A receptor antagonist addition	At higher concentrations of serotonin, the migratory capabilities of cranial neural crest were stimulated. The effect was reversed on mandibular mesenchyme cells	Serotonin and serotonin-1 Areceptor	Mouse embryo	NCCs and mandibular mesenchyme were obtained from E9 and E12 embryos, respectively. Serotonin addition: 10 nM, 100 nM, 1 μM, 10 μM, 100 μM. Receptor Antagonist (10 nM): NAN-190	Moiseiwitsch and Lauder, 1995

In whole mice embryos and cultured frontonasal mass explants, the activation of serotonin-2B receptors promotes cell proliferation in the frontonasal mass (Bhasin et al., 2004) and mandibular mesenchyme cells exposed to serotonin (Buznikov et al., 2001). In mouse calvaria pre-osteoblastic cultured cells (MC3T3-E1), citalopram exposure produces an increase in markers of osteoblastic differentiation (Cray et al., 2014). Similarly, the proliferation rate increases in response to serotonin, and low concentrations of fluoxetine in human-derived induced osteoblast culture, and conversely, high levels of fluoxetine have an inhibitory effect on proliferation (Gustafsson et al., 2006). In ATDC5 cartilage cell line, SSRI treatment upregulates *Sox9* expression, a transcription factor that marks NCCs, and cartilage differentiation (Miyamoto et al., 2017). Interestingly, mice exposed to citalopram *in utero* (E13–E20) exhibit altered calvaria growth and craniofacial anomalies including ectopic sutures, single maxillary incisors, absence of incisor root, and deviated snout (Cray et al., 2014). Another study in mice determined that *in utero* exposure to citalopram increases the risk of craniosynostosis, due to a depletion of Gli1^+^ stem cells and altered homeostasis of the suture mesenchymal cells in the calvaria (Durham et al., 2019; Figure 3 and Table 1).

Other animal models, different from mice and humans, show similar responses to serotonin imbalance. Frogs exposed to fluoxetine have a delay in chondrocranial development (Calibuso-Salazar and Ten Eyck, 2015). In *Xenopus laevis*, the serotonin-2B receptor is the regulator of post-migratory NCCs without altering early steps of migration. Overexpression of this receptor induces ectopic visceral skeletal elements and alters the patterning of branchial arches. Additionally, loss-of-function experiments reveal that this receptor signaling is necessary for the formation of jaw joints and the mandibular arch skeletal elements (Reisoli et al., 2010). Incubation with SSRIs (citalopram and fluoxetine) during zebrafish development decreases bone mineralization and the expression of mature osteoblast-specific markers during embryogenesis (Fraher et al., 2016) (Figure 3, Table 1).

Mandible-forming cells and tooth germ development are also sensitive to fluctuations in serotonin levels. Serotonin exerts its effects through modifying the expression of growth factors, such as IGF-1, which is positively regulated by low-to-medium doses of serotonin, and activation of serotonin-1A and serotonin-4 receptors in micromass mandibular cell cultures (Lambert and Lauder, 1999). In addition, in mandibular micromass cultures and mandibular explants, serotonin and activation of specific serotonin receptors can modulate the extracellular matrix, increasing the expression of aggrecan and inhibiting the production of tenascin, two molecules relevant in craniofacial development (Moiseiwitsch and Lauder, 1997; Moiseiwitsch et al., 1998). In mouse mandibular explants, it has been described that serotonin facilitates the morphological transitions at the early stages of the tooth germ by regulating proliferation rates: whereas low concentrations of serotonin stimulate cell proliferation, high concentrations inhibit proliferation in different areas, shaping the dental epithelium and mesenchyme. Hence, in organ cultures without serotonin, tooth germ develops only up to the bud stage. When the medium is supplemented with serotonin, the cultured explants reach a late bell stage in a dose-dependent manner (Moiseiwitsch and Lauder, 1996). According to that, fluoxetine affects the interaction between epithelium and mesenchyme arresting tooth development at the early stages (Moiseiwitsch et al., 1998). Later, during the initial postnatal days, SSRI reduces the transcription of enamel proteins and secretion of vascular factors in mouse enamel organ and cultured ameloblast-like cells that indicate possible adverse effects of fluoxetine on amelogenesis (Riksen et al., 2010). In adult rats, platelet-derived serotonin has been related to the recruitment of dental stem cells after injury: when platelets come from rats with deficiency of serotonin storage, dentin reparation is impaired (Baudry et al., 2015). All the research presented suggests that the balance of serotonin signaling is important for the correct development of the mandible and teeth, potentially affecting the different developmental processes in which stem/progenitor cells and the differentiation of their progeny are involved (Figure 3 and Table 1).

In the case of the salivary glands, they have a common progenitor with tooth germs generated from the same ectodermal-derived epithelium and NCC mesenchyme (Jimenez-Rojo et al., 2012; Chatzeli et al., 2017; Emmerson et al., 2017) and, therefore, being prone to be affected by a serotonin imbalance. Indeed, fluoxetine treatment modifies the salivary flow rate, mass, and cell volume, indicating its role in adult salivary gland function in rats and humans (Hunter and Wilson, 1995; Turner et al., 1996; da Silva et al., 2009; Henz et al., 2009; Paszynska et al., 2013). Nevertheless, there are no studies showing the role of SSRI in salivary gland formation.

Palate formation has also been associated with serotonin signaling. Interestingly, one recent work indicates that mice exposed *in utero* to sertraline generate significantly more cleft palate than the control group (Cabrera et al., 2020). Thus, it is proposed that serotonin and antagonist of serotonin receptors alter the rotation of the palate shelves in mouse embryo culture (Wee et al., 1979, 1981; Zimmerman et al., 1981). Similarly, cleft lip with or without palate has an increasing risk in mothers that use SSRIs (Louik et al., 2007; Colvin et al., 2011; Malm et al., 2011).

Similar to the craniofacial tissues, serotonin also controls proliferation in the nervous system. Serotonin induces the proliferation of fetal hypothalamic neuroprogenitor cells *in vitro*, demonstrated by the increase in neurospheres and undifferentiated Sox2^+^ stem cells, with a decrease in mature NeuN^+^ neurons (Sousa-Ferreira et al., 2014). Importantly, on the adult dentate gyrus of *Btg1* knock-out mice, characterized by reduced self-renewal and proliferative capability, fluoxetine can reactivate the proliferation of neural stem cells in a similar manner that Sox2 overexpression does in these animals (Micheli et al., 2018) (Table 1). Interestingly, tooth germ, salivary glands, and palate have stem/progenitor cells that are positive for Sox2 and Sox9 transcription factors, which are affected by SSRI in chondrogenic and neural context, suggesting that these cells could be also affected in these organs (Gaete et al., 2015; Kawasaki et al., 2015; Chatzeli et al., 2017; Emmerson et al., 2017).

In conclusion, serotonin levels are associated with the regulation of proliferation, differentiation, and migration of craniofacial tissues and stem/progenitor cells including those that form bone, cartilage, tooth germ, salivary gland, and palate (Figure 3). All these processes are critical for the formation of the craniofacial region and can alter the cellular conditions with an outcome in craniofacial defects (Figure 1). Considering this, depression and antidepressants have the potential of causing craniofacial defects based on the interference in the extensive cellular and developmental process of the embryo (Figure 3).

## Fox Transcription Factors as a Potential Connection Between Serotonin Deregulation and Disrupted Cranial Stem Cell Biology

One interesting goal is to understand how the levels of serotonin are translated into transcription factor expression that leads to changes in proliferation, migration, and differentiation of craniofacial cells. Recently, the Forkhead transcription factor family, characterized by their DNA-binding domain called Forkhead box (Fox), has been associated with craniofacial development.

A modular expression of distinct subclasses of Fox proteins (Foxc/d/f) was observed in the zebrafish facial tissue, linked with important craniofacial signaling pathways like Fgf, Bmp, and Hh among others. Additionally, using TALENs (transcription-activator-like effector nuclease) and CRISPR/Cas9 technologies to generate mutant zebrafish embryos for specific Fox genes, different facial cartilage and tooth defects were detected depending on the specific mutated genes, showing that Fox proteins are required for craniofacial development (Xu et al., 2018). Foxc1 function is required for access to chondrocyte-specific enhancers in zebrafish face; within this subset of cartilage elements, approximately a third of them have Fox and Sox response elements, suggesting that Foxc1 could promote Sox9 binding to those enhancers by increasing chromatin accessibility (Xu et al., 2021). Foxc2 could cooperate with Foxc1 in the development of the cranial base, since both are co-expressed in this area during mouse craniofacial development. Foxc2 silencing through the Cre-recombinant system showed a lack of ossification in the presphenoid, while Foxc1 silencing exhibit a non-ossification of the presphenoid, a deformed alisphenoid, and severe loss in the anterior part of the basisphenoid (Takenoshita et al., 2021). These studies introduce Fox proteins as important players to consider during craniofacial development.

**FIGURE 4:**
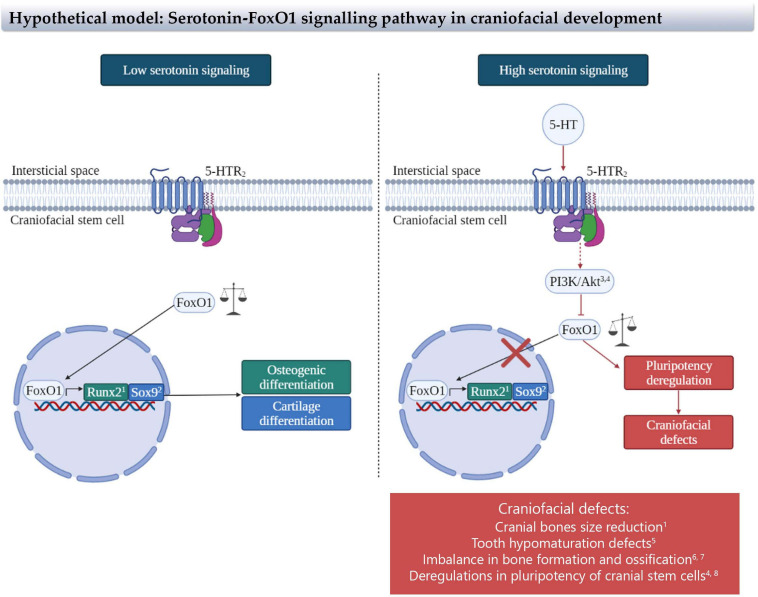
Representation of the proposed link between serotonin and craniofacial defects mediated by FoxO1 transcription factor. Our hypothetical model proposes that under low serotonin signaling, FoxO1 enters to the nucleus generating the transcription of osteogenic (Runx2) and cartilage (Sox9) differentiation genes promoting the differentiation of bones and cartilages. On the contrary, under high serotonin signaling, the activation of the serotonin two receptor, through PI3K/AKT signaling pathway, impedes the entrance of FoxO1 to the nucleus, generating an imbalance in the pluripotency genes related to craniofacial defects. Craniofacial defects observed are included. References: ^1^Teixeira et al., 2010; ^2^Kurakazu et al., 2019; ^3^Lv et al., 2017; ^4^Ormsbee Golden et al., 2013; ^5^Poche et al., 2012; ^6^Almeida et al., 2007; ^7^Iyer et al., 2013; ^8^Zhang et al., 2011.

Among the Fox proteins, the FoxO subfamily transduces environmental signals, affecting gene expression associated with cell proliferation, differentiation, apoptosis, and metabolism, among other processes (Carlsson and Mahlapuu, 2002; Benayoun et al., 2011). In the last years, there has been increasing evidence linking FoxO proteins to the regulation of bone formation (Huang et al., 2020; Ma et al., 2020). It has been demonstrated that FoxO1 functions as an early regulator of osteogenic differentiation in MSCs. FoxO1 silencing leads to a 20% reduction in the size of the mandible, premaxilla, and nasal bones of mice embryos, in addition to a 40% decrease in ossification on the palatine process through direct interaction with Runx2, an important factor in craniofacial bone differentiation (Teixeira et al., 2010). Runx2 has been proposed as a mediator for the gut-derived serotonin suppressive action on the bone formation, with a bimodal action on the tissue. At the physiological circulating serotonin levels, there exists a balance in FoxO1 expression promoting osteoblast proliferation. On the contrary, at high serotonin levels, the balance is disrupted increasing its transcriptional activity that suppresses cell cycle progression genes (Kode et al., 2012; Figure 4). Using *C. elegans*, it was observed that a serotonin deficit promotes nuclear accumulation of Daf-16, a FoxO ortholog (Liang et al., 2006). An enhancement of serotoninergic activity by d-fenfluramine treatment increased the inhibitory phosphorylation of FoxO1 in several regions of the mouse brain (Polter et al., 2009), adding evidence of FoxO regulation by serotonin in the mammalian brain. On the other hand, serotonin can improve hematopoietic stem/progenitor cell survival through the inhibition of the AKT-FoxO1 signaling pathway during embryonic development (Lv et al., 2017).

FoxO1 acts as a pluripotency regulator in embryonic stem cells interacting with Sox2 and Oct4, strong pluripotency regulators, through modulating their expression (Zhang et al., 2011; Ormsbee Golden et al., 2013). Other regulatory actions have been reported for FoxO1 on the craniofacial stem/progenitor marker Sox9. Thus, *FoxO1* knock-down leads to a lower expression of Sox9 in ATDC5 cells (Kurakazu et al., 2019). The same study showed that both FoxO1 and Sox9 start to increase their expression at the same time during chondrogenic differentiation, suggesting that both transcription factors interact to contribute to the differentiation process. This cooperation between both transcription factors has been previously suggested by the identification of Fox consensus binding motifs highly enriched in Sox9-bound enhancers of chondrocytes genes (Ohba et al., 2015; Figure 4).

Furthermore, FoxO genes have the capacity to antagonize Wnt/β-catenin signaling through its association with β-catenin that blocks its interaction with TCF/LEF transcription factors, attenuating bone formation in bipotential osteoblast precursors. This effect has been proposed as a molecular mechanism for the possible loss of periodontal ligament, bone, and tooth derived from periodontal disease (Almeida et al., 2007; Galli et al., 2011; Iyer et al., 2013). Hence, tooth development could be also affected by serotonin-derived FoxO deregulation. Experiments using ameloblast-specific knock-out for *FoxO1* showed mice with enamel hypomaturation defects, resulting in faster attrition of the teeth during mice life (Poche et al., 2012).

Additionally, it has been reviewed that FoxO genes are involved in the regulation of the behavioral manifestation of depression. These proteins are not only expressed in brain areas that respond to emotional stimuli but also are related to circadian rhythm regulation, for which disruptions are associated with major depression (Wang et al., 2015). In a recent study, *FoxO1* mRNA and protein levels were reduced in the prefrontal cortex of depressive postpartum mice induced through chronic unpredictable stress treatments (Liu et al., 2020). Mice with brain knock-out for *FoxO1* display increased depressive behaviors and reduced anxiety (Polter et al., 2009).

Altogether, these studies suggest that FoxO1 could be part of the mechanism involved in the craniofacial defects due to the disrupted serotonin levels present in depressed mothers. Here, we propose a model in which FoxO1 acts as an integrator of the serotonin signaling with the specific stem/progenitor cells involved in the craniofacial development (see Figure 4).

## The Use of Serotonin-Related Antidepressants Increases the Risk of Craniofacial Development Defects in Humans

Although there is sufficient biological basis to establish an association between serotonin deregulation and SSRI use during pregnancy as environmental factors affecting craniofacial normal development, it remains a controversial topic in the clinical field. Various knowledge resources to investigate alterations in craniofacial development patterns such as genome-wide association studies (GWAS), dysmorphology, twin family, and animal and population studies are highly available. The last two approaches are the most suitable for elucidating the association between depression/SSRIs and craniofacial defects.

Prospective cohort investigations have been published with the aim of clarifying the association between antidepressant use during pregnancy and major congenital malformations. Berard et al. (2017) determined the association between first-term exposure to antidepressants and the risk of major congenital malformations in a cohort of depressed/anxious women. These data were obtained from the Quebec Pregnancy Cohort, including all pregnancies diagnosed with depression or anxiety, or exposed to antidepressants in the 12 months prior to pregnancy that ended with a live-born child. When looking at the specific types of antidepressants used during the first trimester, only the SSRI citalopram increased the risk of major congenital malformations [adjusted odds ratio (OR) 1.36, 95% CI 1.08–1.73], although there was a trend toward an increased risk for the most frequently used antidepressants. Regarding the craniofacial territory, citalopram increased the risk of craniosynostosis (adjusted OR 3.95, 95% CI 2.08–7.52), tricyclic antidepressants (TCA) were associated with eye, ear, face, and neck defects (adjusted OR 2.45, 95% CI 1.05–5.72), indicating that antidepressants with effects on serotonin reuptake during embryogenesis increased the risk of some craniofacial malformations in a cohort of pregnant women with depression (Berard et al., 2017). Using the same population-based cohort study in Quebec, the authors concluded that sertraline increases the risk of craniosynostosis (OR 2.03, 95% CI 1.09–3.75) when it is compared with depressed women not using pharmacological antidepressant therapy. In addition, non-sertraline SSRIs were associated with an increased risk of craniosynostosis (OR 2.43, 95% CI, 1.44–4.11) (Berard et al., 2015). In another cohort population study from Northern Denmark, SSRI treatments were associated with an increased risk of malformations (OR 1.3, 95% CI 1.1–1.6) (Kornum et al., 2010). These results were confirmed by a systematic review that analyzed different studies of major congenital malformation cohort populations. In general, the use of SSRIs was associated with an increased risk of overall major congenital anomalies (OR 1.11, 95% CI 1.03–1.19). Similar significant associations were observed using maternal citalopram exposure (OR 1.20, 95% CI 1.09–1.31), fluoxetine (OR 1.17, 95% CI 1.07–1.28), and paroxetine (OR 1.18, 95% CI 1.05–1.32) (Gao et al., 2018).

In a case-control study (major birth defects vs. control) using an expanded dataset from the National Birth Defects Study of the United States population, the mothers of the children were asked about the use of antidepressants during the first trimester of pregnancy. Maternal SSRI consumption was associated with craniofacial defects: anencephaly (adjusted OR 2.4, 95% CI 1.1–5.1) and craniosynostosis (adjusted OR 2.5, 95% CI, 1.5–4.0) (Alwan et al., 2007). These results were confirmed by the systematic review, determining an increased odds ratio for birth defects with paroxetine (anencephaly OR 3.2, 95% CI 1.6–6.2) and fluoxetine (craniosynostosis OR 1.9, 95% CI 1.1–3.0) (Reefhuis et al., 2015).

Most clinical studies have the difficulty to separate the effects of the underlying depression and the use of antidepressants. One control-case study that considers this variable, comparing the offspring defects of women with unmedicated depression, women with treated depression and women without depression, determined that compared with women without depression, major congenital anomalies were not associated with unmedicated depression (adjusted OR 1.07, 95% CI 0.96–1.18), SSRIs (adjusted OR 1.01, 95% CI 0.88–1.17), or TCAs (adjusted OR 1.09, 95% CI 0.87–1.38) (Ban et al., 2014). A previous work found an increased risk of major congenital anomalies in infants born from women who took SSRIs in the first trimester of pregnancy (adjusted OR 1.33, 95% CI 1.16–1.53), whereas the correlation was not significant for women who paused their SSRI intake (adjusted OR 1.27, 95% CI 0.91–1.78) (Jimenez-Solem et al., 2013). This issue was considered by the systematic review of Gao et al. (2018), in which they studied a population of women with a psychiatric diagnosis (depression or anxiety) as a different group of comparison. No significantly increased risk was observed in this group compared with the control group (major congenital anomalies, OR 1.04, 95% CI 0.95–1.13) (Gao et al., 2018). From these studies, we can infer that depression itself is not a risk factor for congenital anomalies. However, more research is still necessary to conclude this.

Despite the limitations and the different results between the cited studies, they all share the conclusion that SSRI usage during the first trimester of pregnancy is associated with a higher risk of congenital malformations and specifically craniofacial defects, in which a higher risk of craniosynostosis and other defects with some SSRIs are reported. All the studies presented here emphasized that an increasing number of women with depression during pregnancy is being diagnosed and that the use of SSRIs has been increased in the past years. Thus, it is important that to review its use in pregnant or reproductive-age women. Therefore, these results should have direct implications on their clinical management.

## Future Perspectives

**FIGURE 5:**
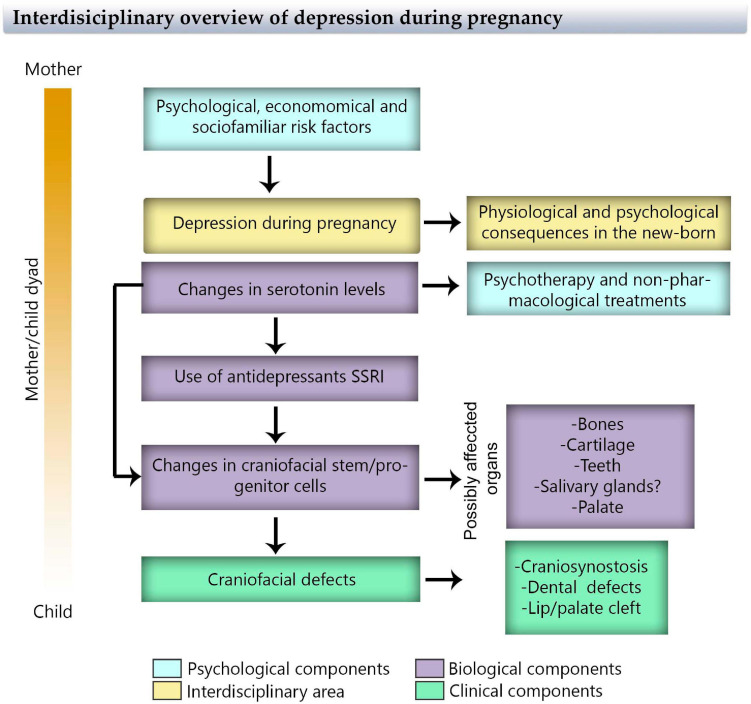
Flowchart of maternal depression during pregnancy through an interdisciplinary view considering psychological, physiological, biological, and clinical components that affect the mother/child dyad.

The treatment of maternal depression during pregnancy with a combination of psychotherapy and antidepressants is widely used, however, some studies reveal negative effects: The use of SSRI antidepressants increase the risk of congenital craniofacial defects in the newborn. It is important to highlight that maternal depression impacts the mother–fetus/baby dyad, and this should be considered during prevention, diagnosis, and treatment. An interdisciplinary approach that considers biological, clinical, psychological, social, and familiar aspects is also fundamental (Figure 5). New treatments should include the provision of a support network and the identification of lifestyle risk factors that may contribute to maternal depression (diet, physical activity, etc.), and weigh the possibility of adherence and prejudices about the selected treatment.

**FIGURE 6:**
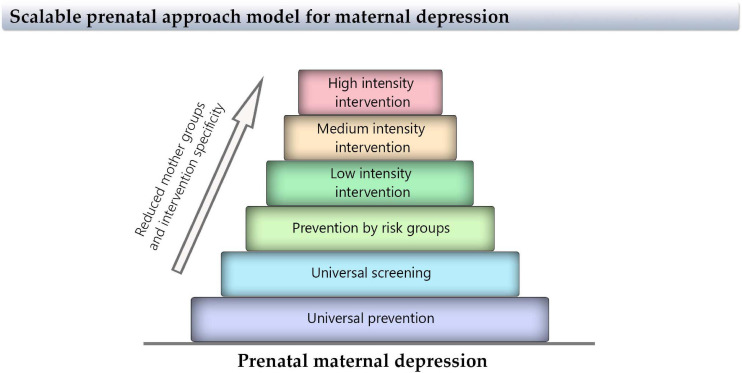
Scale of proposed approaches for maternal depression interventions. This pyramid represents the size of the interventions for maternal depression, indicating that the basis should be attended wide and escalating over more specific groups and therapies.

The application of early preventive intervention programs that increases wellness and promotes the mental health of mothers and their children, considering that the previous history and habits of the mother is of major relevance. To take the best decision for the treatment for each dyad, it is necessary to perform more longitudinal studies that consider the time and comorbidities of maternal depression and the impact on the offspring. These methods could include the implementation of scalable prenatal approach models: universal prevention→universal screening→prevention indicated for risk groups: early low-, medium-, and high-intensity specialized intervention (all based on evidence). This model can be represented as a pyramid to understand that the basics should be attended widely, escalating over more specific groups and therapies (Figure 6). Additionally, it is important to promote high-quality research in innovative treatments for depression, for instance, food supplements (Sparling et al., 2017) or transcranial magnetic stimulation (Kim et al., 2019). Finally, we think it is important to build, review, and recommend “decision aid protocols” to analyze individually the risk–benefit balance of the antidepressant treatment. Nowadays, this is of particular importance when the percentage of depressive women has increased over the last years (Global Burden of Disease Study, 2017).

As we described in this review, the craniofacial region appears to be especially sensitive to changes in serotonin signaling, where the imbalance generates defects in bone development, cartilage maturation, tooth germ, and palate formation. Even the effects of depression itself appear to be marginal in epidemiological studies; the use of SSRI that cross through the placenta (Rampono et al., 2009) could affect the development of the fetus, increasing the risk of craniofacial defects. Interestingly, at the time when the craniofacial region is actively forming, most women are not aware of being pregnant, so if they are using an SSRI, probably they will continue to do so. Thus, the decision to prescribe antidepressant medication during pregnancy or on fertile women must be weighed against the risks of untreated maternal depression.

In our revision, we found limited research about biological aspects linking serotonin and craniofacial defects, indicating a great necessity to investigate this topic. This gained special relevance in light of the prevalence and clinical problems that the deregulation of serotonin implies during development. Importantly, most of the publications in the biological field are from decades ago, so the advantage of cutting-edge methods is not employed. New experimental models can be used to explain the underlying mechanisms of clinical problems related to craniofacial defects, including genetic and/or environmental etiology. Models like zebrafish and frog larva offer a great possibility to test not only pollutants such as bisphenol but also drugs such as SSRI, by adding them directly into the liquid growth medium, allowing to test different doses and drugs (fluoxetine, citalopram, and sertraline, etc.) (Calibuso-Salazar and Ten Eyck, 2015). Organ culture also offers similar advantages to test diverse doses and drugs by adding them directly to the culture medium (Sánchez et al., 2018). These experiments can provide valuable information about serotonin regulation in a quick and easy manner, which then can be complemented with *in vivo* studies using, for example, mice models for depression (Krishnan and Nestler, 2011; Planchez et al., 2019) or using SSRIs during pregnancy in murine models.

Here, we mentioned that the mechanism by which serotonin deregulation could affect craniofacial development is not totally elucidated, but that this includes developmental biology processes and the biology of stem/progenitor cells. Accordingly, NCCs and MSCs appear to be the most affected cells, especially given their proliferative and migrative capacities that impact over facial and skull bone, cartilage, palate, and tooth formation. The similarities between the tooth and salivary gland formation, and the influence of serotonin in the neural-crest derived mesenchyme, make evident the necessity of future studies about how serotonin deregulations could affect salivary gland development.

In this review, we propose that the transcription factor FoxO1 could be implicated linking the misregulation of serotonin levels with the different processes affected during craniofacial formation, disrupting the stem/progenitor cell biology. FoxO1 has a role in craniofacial tissue development (bone, cartilage, and tooth) and function within the stem cell regulation (Xu et al., 2018, 2021; Takenoshita et al., 2021). Moreover, it has a capacity to respond to changes in serotonin concentrations, being involved in the manifestation of major depressive disorders (Polter et al., 2009; Wang et al., 2015). All these together suggest that FoxO1 functions as a potential link between the craniofacial development and disrupted serotoninergic signaling by a specific context/environment. However, more studies are necessary to determine this hypothesis and the mechanism disrupted by the changes in serotonin levels by depression or SSRI use.

Regarding the clinical research field, we need to consider some limitations, such as difficulties on patient recruitment, withdrawal of patients, insufficient statistical power, issues with the classification of birth defects, the presence of confounders, or poor information about the medication exposure. Additionally, it is difficult to determine an SSRI dose-response effect because, in general, the information comes from maternal reports that are imprecise. A similar difficulty occurs with the type of antidepressants because the mothers were usually asked about the commercial name of the drug, generating an under- or over-representation of some antidepressants and possible bias in the responses. Another important limitation is the presence of confounders such as smoking, folic acid, alcohol, or other drug intakes that are commonly present in the lifestyles of mothers with depression. Although the study design tried to consider these variables, it is not completely reliable given that these depends on patient reports.

Randomized clinical trials contrasting a group of depressed pregnant women with and without pharmacological treatment would allow us to further elucidate the relationship between antidepressants and congenital malformations. Currently, a randomized placebo-controlled trial on depressed mothers is being conducted in Stockholm; the results in the child exposed or not exposed to SSRI *in utero* will be analyzed (Heinonen et al., 2018). In this way, while current systematic reviews (Uguz, 2020) of meta-analyses examining the relationship between maternal use of SSRI during pregnancy and congenital anomalies have suggested a significant positive association between the use of SSRIs and the risk of major congenital anomalies, further large-scale prospective observational studies, and meta-analyses on the effects of SSRIs are required to reach definitive conclusions. However, since risk estimates for adverse events are similar in randomized trials and observational studies, the findings described in this review have implications for clinical practice (Golder et al., 2011).

In conclusion, serotonin appears to be involved in many developmental processes and the deregulation of its signaling, and the use of SSRI antidepressant leads to an increased risk of craniofacial development defects. Maternal depression during pregnancy needs to be carefully treated, diminishing the use of pharmacotherapy, and highlighting psychotherapy and alternative tools for the treatment, especially in minor and middle depression. Serotonin can affect the balanced role of NCCs and MSCs, but more research is necessary to determine the mechanism by which serotonin could influence the development of craniofacial tissues with special attention to stem/progenitor cells, aiming to discover alternative pathways to prevent the craniofacial development defects generated.

## Author Contributions

NS, JJ-B, and MG reviewed the literature, wrote the developmental biology sections of the review, and formulated the figures and tables. MO, AM, MM, and PF reviewed the literature and wrote the psychological sections of the review. HG-O and FB reviewed the literature and wrote the clinical section of the review. NS and MG edited all the sections. All authors contributed to the discussion of the document.

## Conflict of Interest

The authors declare that the research was conducted in the absence of any commercial or financial relationships that could be construed as a potential conflict of interest.

## Publisher’s Note

All claims expressed in this article are solely those of the authors and do not necessarily represent those of their affiliated organizations, or those of the publisher, the editors and the reviewers. Any product that may be evaluated in this article, or claim that may be made by its manufacturer, is not guaranteed or endorsed by the publisher.
